# Initial evaluation of extracorporeal immunomodulatory therapy for the treatment of critically ill COVID-19 infected patients

**DOI:** 10.1038/s41598-022-21944-4

**Published:** 2022-11-24

**Authors:** Sandrine Lemoine, Jarrin Penny, Douglas D. Fraser, Fabio R. Salerno, Justin Dorie, Tanya Tamasi, Robert Arntfield, Andrew House, Marat Slessarev, Christopher W. McIntyre

**Affiliations:** 1 The Lilibeth Caberto Kidney Clinical Research Unit (KCRU), Kidney Clinical Research Unit Room ELL-101, London Health Sciences, University of Western Ontario, Centre 800 Commissioners Rd E, London, ON N6A5W9 Canada; 2 Robarts Research Institute, Western University, London, Canada; 3 Department of Medical Biophysics, University of Western Ontario, London, ON Canada; 4 Division of Nephrology, Schulich School of Medicine & Dentistry, Western Ontario, London, Canada; 5 Lawson Health Research Institute, London, ON Canada; 6 Departments of Critical Care Medicine, Victoria Hospital, London, Canada; 7 Departments of Pediatrics, Clinical Neurological Sciences and Physiology and Pharmacology, Western University, London, ON Canada

**Keywords:** Randomized controlled trials, Renal replacement therapy

## Abstract

Severe COVID-19 infection results in significant immune dysregulation resulting from excessive recruitment and activation of neutrophils. The aim of this study was to confirm feasibility, initial safety and detect signal of efficacy of a non-propriety device delivered using an intermittent extra-corporeal system (LMOD) allowing leucocytes modulation in the setting of Severe COVID-19 infection. Twelve patients were recruited. Inclusion criteria were > 18 years age, confirmed COVID-19, acute respiratory distress syndrome requiring mechanical support and hypotension requiring vasopressor support. Primary end point was vasopressor requirements (expressed as epinephrine dose equivalents) and principle secondary endpoints related to safety, ability to deliver the therapy and markers of inflammation assessed over five days after treatment initiation. LMOD treatment appeared safe, defined by hemodynamic stability and no evidence of white cell number depletion from blood. We demonstrated a significant decrease in vasopressor doses (−37%, *p* = 0.02) in patients receiving LMOD therapy (despite these patients having to tolerate an additional extracorporeal intermittent therapy). Vasopressor requirements unchanged/increasing in control group (+ 10%, *p* = 0.48). Although much about the use of this therapy in the setting of severe COVID-19 infection remains to be defined (e.g. optimal dose and duration), this preliminary study supports the further evaluation of this novel extracorporeal approach.

## Introduction

Clinical features of COVID-19 vary widely between patients; from mild respiratory tract illness to severe progressive interstitial pneumonia, multiorgan failure, and death^1^. Some patients develop systemic hyperinflammation syndrome associated with shock, vasoplegia, respiratory failure, and cardiopulmonary collapse. Severe COVID-19 infection results in significant immune dysregulation resulting in a proinflammatory cytokine storm, and ultimately a proteolytic storm, resulting from excessive recruitment and activation of neutrophils. There is still a dearth of effective and widely available therapeutic options for the management of the most severely critically ill patients^2,3^. An urgent need exists for additional non-proprietary therapeutic approaches, that can be rapidly deployed in a cost-effective manner, effectively utilizing resources already widely available in the critical care setting.

Preliminary study has confirmed effectiveness of a leukocyte modulation device (LMOD) in the setting of sepsis. Activated leukocytes present in the blood are sequestered onto a fiber surface, immunomodulated in this specialized environment and released back into circulation. This process aims to mitigate the pro-inflammatory response by altering the ratios of leukocyte subpopulations (macrophages and neutrophils), and influencing the downstream activities of immune effector cells^4^. A proprietary LMOD has been previously described (although currently not commercially available). Preliminary study has demonstrated efficacy in a pig model of sepsis. Subsequentley, the LMOD has been demonstrated to ameliorate multi-organ dysfunction in four ICU based clinical trials, resulting in a relative 50% reduction in mortality rates^5,6^. A preliminary case report (published after our current study had commenced) suggested modulation of inflammatory response associated with severe COVID-19 infection after incorporating a LMOD device within an ECMO circuit in two patients^7^.

We hypothesized immunomodulation using the LMOD in patients with severe COVID-19 will result in the attenuation of circulating cytokine levels and be associated with improved clinical status. The aim of this initial proof of principal short-term study was to confirm feasibility, initial safety and detect signal of efficacy of a non-propriety device delivered using an intermittent extra-corporeal system.

## Results

### Patient characteristics

We included 12 patients. Patient characteristics are described in Table 1. There were no differences between basal characteristics between groups. No patients received neither antiviral treatment nor hydroxychloroquine. All patients (LMOD and control group) have been treated with dexamethasone 6 mg daily during the follow up.

**Table 1:** Patient’s characteristics.

Median (IQR)	LMOD group n = 6	Control group n = 5
**Patient’s characteristics**		
Age (years)	59 (54–66)	69 (55–74)
Male, n (%)	4 (66%)	4 (80%)
BMI (kg/m^2^)	30.5 (27.4–34.4)	31.6 (27.5–39.4)
Days before worsening	6 (6–11)	5.5 (4–11)
Hypertension, n (%)	5 (83%)	2 (40%)
Diabetes T2, n(%)	2 (33%)	3 (60%)
SOFA at D1	13.5 (12.25–15.25)	11 (10–14)
**Ventilation**		
FIO2 (%)at D1	80 (69–89)	65 (49–72)
PEEP at D1	16 (16–17.5)	16 (10–18)
P/F ratio at D1	102 (86.5–148.5)	100 (92–172)
PCMV = pressure control mechanical ventilation, n(%)	4 (66%)	3 (50%)
Volume control at D1	16 (14–20)	14 (14–24)
Tidal volume at D1	400 (347–446)	389 (377–407)

BMI = Body mass index, SOFA = Sepsis-related Organ Failure Assessment, WC = white blood cells, CRP = C-reactive protein, AST = Aspartate aminotransferase.

*Friedman test, p significant for *p* < 0.05.

### Feasibility and safety

**Figure 1:**
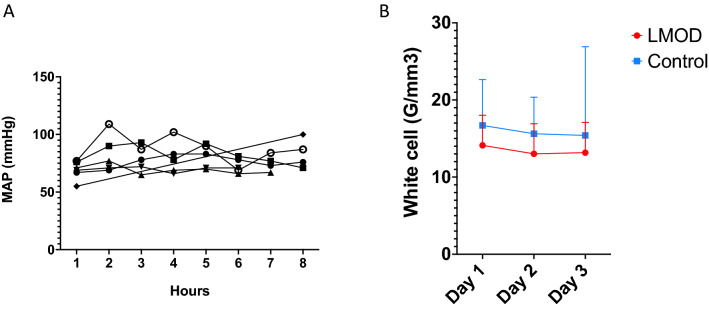
Safety of LMOD treatment. (**A**) median of the mean arterial pressure all the LMOD treatment along. (**B**) Whites cells changes in LMOD group compared to control group. L-MOD patients are in red and control patients in blue. MAP = Mean arterial blood pressure.

All treatments were successfully delivered, without the requirement for early termination or loss of the circuit due to thrombosis. LMOD treatment appeared safe, defined by hemodynamic stability (Fig. 1A) and no evidence of white cell number depletion from blood (Fig. 1B).

Median (IQR) ionized calcium before the LMOD was 0.38 (0.34–0.40, 100% of patients had an iCa < 0.40 mmol/L. Median (IQR) ionized calcium to the patient was 1.13 (1.08–1.2) mmol/L. No patient experienced hypo or hypercalcemia, thrombocytopenia or allergic reaction.

### Primary outcome (Table 2)

**Table 2:** Safety and outcomes.

Median (IQR)	LMOD group n = 6	Control group n = 5
**Primary outcome and safety**		
Vasopressor support (epinephrine dose equivalent) at day 1	16 (8.7–25)	10 (3.5–20)
Vasopressor support (epinephrine dose equivalent) at day 5	14.5 (8–20) *	16 (4.5–45)
WC at D1 (G/L)	13 (11–17)	19 (11–21)
WC at D5 (G/L)	15 (9–16)	14 (5–27)
Lymphocytes at D1 (G/L)	0.8 (0.6–1.4)	1.1 (0.6–2)
Lymphocytes at D5 (G/L)	1 (0.5–1.8)	0.65 (0.5–1.2)
Thombocytes at D1 (G/)	221 (168–260)	266 (232–280)
Thombocytes at D5 (G/L)	156 (145–247)	251 (220–395)
**Secondary outcomes**		
SOFA at D1	13.5 (12.25–15.25)	11 (10–14)
SOFA at D5	11.5 (10–13.5)	12 (9–13)
CRP at D1 (mg/L)	186 (44–306)	63 (29–220)
CRP at D5 (mg/L)	125 (51–208)	85 (17–163)
Ferritin (umol/L) D1	2061 (1088–3268)	1174 (793–3555)
Ferritin (umol/L) D5	1096 (495–3670) *	823 (710–1650)
D-Dimere (μg/L) D1	7706 (2684–15,694)	4025 (2088–8460)
D-Dimere (μg/L) D5	3559 (2210–7038)	2340 (2081–2738)
Troponin (ng/L) D1	61 (27–640)	38 (14–115)
Troponin (ng/L) D5	43 (20–517)	47 (17–90)
AST at D1 (U/L)	48 (37–107)	48 (40–80)
AST at D1 (U/L)	45 (36–118)	56 (49–65)

SOFA = Sepsis-related Organ Failure Assessment , WC = white blood cells, CRP = C-reactive protein, AST = Aspartate aminotransferase.

*Friedman test, *p* significant for *p* < 0.05.

**Figure 2:**
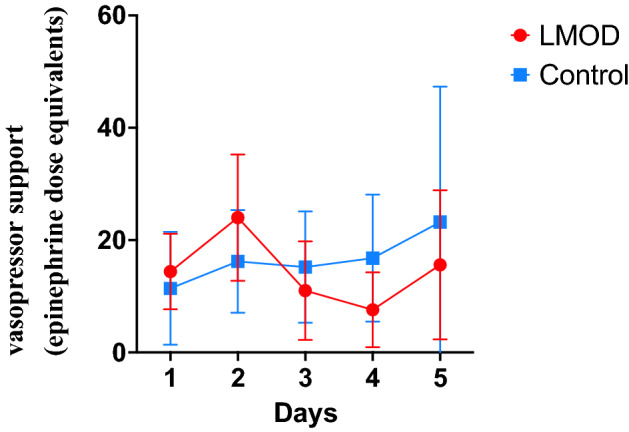
Primary outcome. Vasopressor support changes during the 5 days of follow-up. L-MOD patients are in red and control patients in blue. We showed a significant decrease of vasopressor drugs (*p* = 0.02, Friedman test), and a trend to drugs dose for control patients.

We demonstrated a significant decrease in vasopressor doses (−37%, *p* = 0.02) in patients receiving LMOD therapy (despite these patients having to tolerate an additional extracorporeal intermittent therapy). Vasopressor requirements unchanged/increasing in control group (+ 10%, *p* = 0.48) (Fig. 2).

### Secondary outcomes (Table 2)

**Figure 3:**
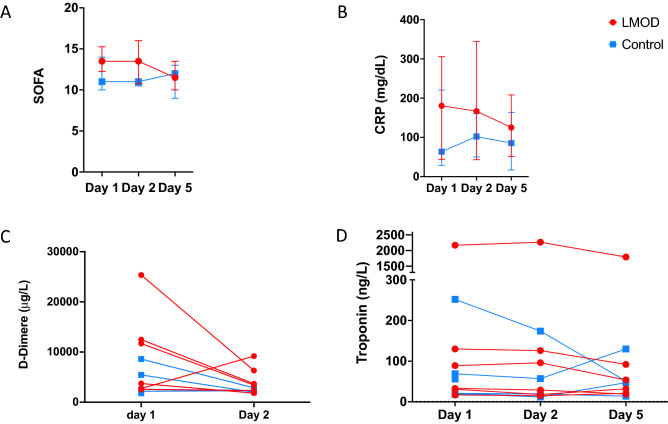
Secondary outcomes. Changes of (**A**) SOFA score. (**B**) CRP (**C**) D-Dimer (**D**). Troponin. SOFA = Sepsis-related Organ Failure Assessment, CRP = C-reactive protein.

At day five, we saw no deaths in treated group vs. 2/6 deaths in the control group. Median SOFA score was 13.5 at Day 1 with a sustained reduction after LMOD treatment (day five median 11.5, *p* = 0.08; in contrast to unchanged SOFA scores in the control group^6^ (11 at baseline and 12 at day five) (Fig. 3A). We found no significant differences between L-MOD and control group with respect to CRP (180 (33–305 mg/dl) vs 63 (29–220) mg/dL, *p* = 0.39 for treated vs control group respectively) (Fig. 3B). We observed a non-significant decrease in CRP levels in the treated group (Day five, CRP 125 (51–208) mg/dl, p = 0.56). Ferritin level was not different at inclusion although we had a trend for a higher level in treated group (2061 (1088–3268)) vs 1174 (793 -3555), *p* = 0.48). However, there was a significant decrease of ferritin between baseline and Day five only in the treated group (*p* = 0.03) vs control (*p* = 0.25) (Fig. 3C). We found a trend to a larger decrease of cTnT levels in L-MOD group (Fig. 3D).

### Cytokines measurements (Table 3)

**Table 3:** cytokine’s measurement.

	IL-1RA	IL6	TNFα	IL10	IL8	M_CSF	Elastase
LMOD group D1	1614 ± 3793 (*p* = 0.06)	2512 ± 3159	95 ± 60	42 ± 28 Ϯ	139 ± 83 *Ϯ	292 ± 174 (*p* = 0.06)	48 ± 21*
LMOD group D5	58 ± 83	824 ± 1079	64 ± 24	36 ± 16	118 ± 57	152 ± 75	25 ± 8
Control group D1	254 ± 192	1220 ± 2530	61 ± 31	96 ± 120	49 ± 42	137 ± 91	35 ± 27
Control group D5	68 ± 88	93 ± 92	3582 ± 2618	35 ± 20	48 ± 12	65 ± 49	12 ± 3

*p < 0.05 LMOD group D1 vs D5 Friedman test.

*|p* < 0.05 control group D1 vs D5.

Ϯ *p* < 0.05 LMOD group D1 vs control group D1.

We showed a significant decrease of IL8 (*p* = 0.03) and Elastase (*p* = 0.03) between D1 and D5 in the LMOD group, and we almost reached a significant difference in IL-R1 and M_CSF in the LMOD group (*p* = 0.06). We found no difference between D1 and D5 in the control group. Although there is no difference, all TNFα measurements increased in the control group and decrease in the LMOD group.

## Discussion

This is the first report of leukocyte modulation delivered using an intermittent treatment regime and extracorporeal system constructed from modified non-proprietary components, to treat COVID-19 in patients requiring mechanical ventilation and vasopressor support. This preliminary study demonstrated that the treatment is feasible (all treatments delivered as planned) and safe (no significant treatment related adverse events, no treatments terminated early due to lack of tolerability). There appears to be a strong initial signal of biological effect using the leukocyte modulation device (LMOD) with respect to the pre-defined primary outcome of pressor dose.

Patients selected as suitable for this study were all very severely affected with multiple organ failure and a high probability for death. The study was not designed to have a significant impact on survival, particularly as treatment was limited to two sessions within 48 h. We were able to show safety of this LMOD despite the severity of the disease being treated. There was no evidence of treatment related thrombosis or hypocalcemia; and we did not encounter any significant difficulties related to recruitment or delivery of the therapy.

There was a significant signal of benefit; including a significant decrease of the vasopressor drugs c.f. no changes at all in the control group and a variety of secondary blood-based endpoints related to the severity of the underlying inflammatory and prothrombotic status.

The other signal that this paper show is the trend to decrease cytokines and inflammatory markers in the LMOD group without trend on the control group.

The main limitation of this report is the small numbers of patients meaning any results should be considered cautiously.

We have chosen to publish the first 12 patients at this early stage after discussion with our DSMB. This was a predefined time point for an initial analysis and readjustment of the sample size based upon evidence of treatment futility or effect size. The primary end point was defined a priori as being reduction in pressor requirements and improvements in hemodynamics. The short treatment interval (two days of therapy) was considered to be potentially too short to meaningfully impact survival, but would allow preliminary safety/feasibility to be assessed and an early indication of potential efficacy. No data to meaningfully inform sample size existed and a convenience sample of 20 was settled upon. An initial analysis after the first three blocks of randomizations was defined a priori, to allow reconsideration of sample size and whether or not modification of the protocol to increase the number of treatments each patient might receive would be appropriate. The decision reflected the terrible burden that COVID 19 infection at this time, with the lack of additional therapeutic options for the most severely affected patients and the increasingly disproportionate stress being felt by healthcare systems in less developed countries. Countries in South Asia, Africa and Latin America possess critical care services, but remain unlikely to have access (or resourcing) to allow access to kind of novel therapeutic approaches, being developed in Europe and North America. LMOD therapy utilizes exclusively non-proprietary components that are readily available globally at modest cost. The extracorporeal circuit itself is maintained on a conventional hemodialysis platform, delivered in a way similar to SLEDD, and is familiar enough for adoption by any ICU that would normally also deliver renal replacement therapy. All required infusates are already in common use in the overwhelming majority of ICUs. It is compatible will all current therapeutic options, aiming to be complimentary and targeting the last and most severe end of this disease spectrum when the cytokine storm has been supplemented by a proteolytic one (neutrophils largely responsible). We have provided links to a detailed description of the construction of device, circuit and operation using a hemodialysis monitor in the supplemental data.

## Conclusion

Although much about the use of this therapy in the setting of severe COVID-19 infection remains to be defined (e.g. optimal dose and duration), this preliminary study supports the further evaluation of this novel extracorporeal approach. Furthermore, this therapy potentially expands the available armamentarium with a treatment that can be delivered within an existing model of care (similar to renal replacement therapy) and by current staff using low-cost non-proprietary components potentially- even within an austere resource setting.

## Methods

### Patients

Twelve patients were recruited, data are reported for 11 as one of the control patients unfortunately died within two hours after being enrolled.

Inclusion criteria were > 18 years age, confirmed COVID-19 PCR positivity, acute respiratory distress syndrome requiring mechanical support and hypotension requiring vasopressor support. Patients were only excluded on the basis of pregnancy, previous evidence of immunosuppression and contraindication to citrate exposure. An initial analysis after the first three blocks of randomizations was defined a priori, to allow reconsideration of sample size and whether or not protocol amendment to increase the number of treatments delivered to each patient might be appropriate.

This study was approved by an institutional review committee (Health Sciences Research Ethics Board at the University of Western Ontario- HSREB11484) and that the subjects gave informed consent. The study was conducted according to the GCP/ICH guidelines and the Declaration of Helsinki (clinical trial number NCT04353674).

### Study design

**Figure 4:**
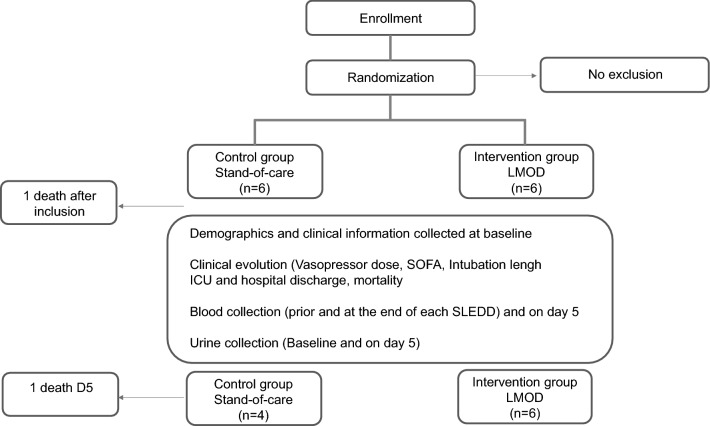
Flow chart of the study.

The flow chart is described in Fig. 4. We performed a 12-patient randomized clinical trial allocating patients (block randomized in groups of four) to either standard care or standard care in addition to LMOD therapy. Study was performed within both main ICUs (Victoria and University Hospitals) within London Health Care Sciences Centre in London, Ontario. Patients received daily 6–8 h LMOD treatments, on two consecutive days.

Primary end point was vasopressor requirements (expressed as epinephrine dose equivalents) and principle secondary endpoints related to safety, ability to deliver the therapy and markers of inflammation assessed over five days after treatment initiation.

### LMOD developpement

**Figure 5:**
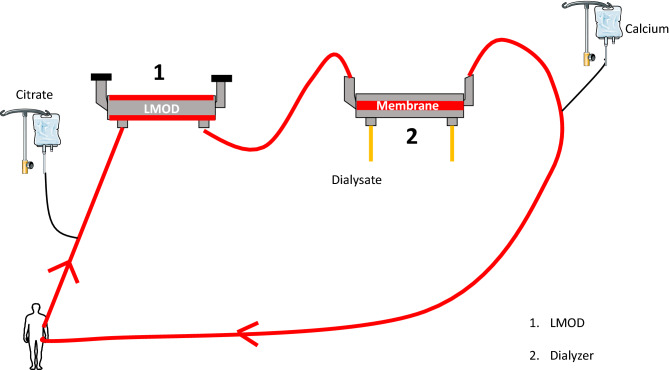
LMOD design schematic.

We rapidly developed a LMOD device for intermittent use (6–8 h treatment) for patients with severe multi-system manifestations of COVID 19 infection, both with and without AKI. We choose this duration of treatment based on practical experience of SLEDD therapy and resource limitations. This was manufactured in house using a standard polysulfone hemodialyzer (Fresenius FX80) with blood flowing through the dialysate compartment. Extracorporeal circuit was maintained using a conventional hemodialysis monitor (Evodial, Baxter Healthcare). The LMOD was anticoagulated using regional citrate, blood then passed through a second dialyzer allowing removal of the infused citrate and repletion of calcium by self-regulating mass transfer from high calcium containing dialysate. Full calcium repletion was achieved by the circuit running in post dilution hemodiafiltration mode (as described previously^8^). Ionized calcium aim was < 0.4 mmol/L before the LMOD. Details concerning sourcing and repurposing of all components, manufacturing details and full description of technical operation are contained in attached linked video. Figure of the circuit is described in Fig. 5.

### Cytokines measurements

Levels of 7 inflammatory analytes were determined using multiplexed biomarker immunoassay kits according to manufacturers’ instructions (MilliporeSigma, 400 Summit Drive, Burlington, MA). Plasma inflammatory analytes were measured using a Bio-PlexTM 200 Suspension Array system (Bio-Rad Laboratories, Hercules, CA), which used Luminex xMAPTM fluorescent bead-based technology (Luminex Corp, Austin, TX).

### Statistical analysis

Descriptive statistics were reported as median [interquartile range (IQR)] for continuous variables and as frequency and percentages for categorical variables. Comparisons between groups were performed using Mann and Whitney test. Fisher’s exact tests were used to compare categorical variables and Friedman test were used for analysis of repeated paired measurements.

Statistical significance was defined as *p* < 0.05. Statistical analysis was performed using GraphPad software (GraphPad Prism 9.1.2 La Jolla, CA).

## Data Availability

The datasets generated during and/or analysed during the current study are available from the corresponding author in reasonable request. However we provide one video to understand the construction of the LMOD. The first covers in detail the in house construction of the LMOD and accompanying circuit, including clear description of the origin and handling of all component parts.: https://drive.google.com/file/d/1gvlEQPtfmbf3xpoozCS4wtFpS55lO5A5/view?usp=sharing
